# Is global health truly global? A hashtag analysis of #GlobalHealth disparities on X

**DOI:** 10.3389/fpubh.2024.1413556

**Published:** 2024-11-29

**Authors:** Zara Arshad, Pranav Sharma, Shweta Sharma, Mustafa Sajjad Cheema, Charu Agarwal, Tamara Tango, Farhan Bin Matin, Atanas G. Atanasov, Bodrun Naher Siddiquea, Maima Matin, Olga Adamska, Artur Stolarczyk, Rahul Kashyap, Faisal A. Nawaz

**Affiliations:** ^1^Global Remote Research Scholars Program, St. Paul, MN, United States; ^2^Lahore Medical College and Institute of Dentistry, Lahore, Pakistan; ^3^Sri Sidhartha Medical College, Tumkur, Karnataka, India; ^4^Faculty of Medicine Universitas Indonesia, Jakarta, Indonesia; ^5^Department of Pharmacy, East West University, Aftabnagar, Dhaka, Bangladesh; ^6^Ludwig Boltzmann Institute Digital Health and Patient Safety, Medical University of Vienna, Vienna, Austria; ^7^Institute of Genetics and Animal Biotechnology of the Polish Academy of Sciences, Jastrzebiec, Magdalenka, Poland; ^8^Laboratory of Natural Products and Medicinal Chemistry (LNPMC), Center for Global Health Research, Saveetha Medical College and Hospital, Saveetha Institute of Medical and Technical Sciences (SIMATS), Chennai, India; ^9^Department of Epidemiology and Preventive Medicine, School of Public Health and Preventive Medicine, Monash University, Melbourne, VIC, Australia; ^10^Faculty of Medicine, Collegium Medicum, Cardinal Stefan Wyszynski University, Warsaw, Poland; ^11^Orthopaedic and Rehabilitation Department, Medical University of Warsaw, Warsaw, Poland; ^12^Department of Research, WellSpan Health, York, PA, United States; ^13^Emirates Health Services, Al Amal Psychiatric Hospital, Dubai, United Arab Emirates

**Keywords:** global health, global health equity, global health discrepancy, hashtag, X, Twitter social media

## Abstract

**Background:**

X (Formerly known as Twitter) healthcare hashtags are a popular healthcare informatics and educational tool among medical professionals. #Globalhealth is one such widely used hashtag with extensive engagement. This study analyses #GlobalHealth to understand its pattern, global digital distribution, and other parameters during the COVID-19 pandemic on X.

**Methods:**

Data was collected by utilizing posts using #GlobalHealth on X from 1st December 2019 to 1st November 2022. The analysis was performed using Symplur Signals to assess several parameters, such as the cumulative number of posts, impressions, category of users, co-occurring hashtags, and geolocation. The Symplur Rank system was used to assess the impact of influencers using the hashtag.

**Results:**

A total of 843,762 posts were shared by 150,408 X users, with 4,639,144,304 impressions. Most posts (73.8%) were made by unclassified accounts, followed by doctors (4.2%), followed by other health workers. The #COVID19 was the most common co-occurring hashtag (43%). The top locations and the most influential X users came from the United States, the United Kingdom, and Canada. Among the top 25 most influential handles, a maximum (*N* = 09) were based in the United States—most profiles (*N* = 10) were categorized as international organizations followed by journals (*N* = 03).

**Conclusion:**

The study gives a glimpse into the discrepancies in global distribution and stakeholders of #GlobalHealth. Most posts originated from the global north, which hints at how the trend to #GlobalHealth is not perhaps as global as it is thought to be, and it also reflects upon the real-world scenarios in the context of Global Health Equity. Thus, deeper and wider studies on this digital discrepancy may add more to the existing discourse on the topic.

## Introduction

The idea of global health reflects a collaborative transnational effort to promote health worldwide. It emphasizes a multidisciplinary approach, where discussions and collaborations are critical to addressing health issues that transcend national boundaries affecting populations across the globe (1). A key aspect of global health involves advocacy and discussions to drive impact and collaborate efficiently.

Many such global health-related discussions and information sharing occur on social media, such as on the popular platform X, where the hashtag “#GlobalHealth” collects and classifies posts related to Global Health. Despite being a common hashtag, it has not been analyzed previously in the literature regarding its digital characteristics and usage. This hashtag is a convenient way for users to engage in global health dialog where multiple stakeholders participate, including individuals and organizations, healthcare professionals, researchers, advocacy groups, and journalists. This leads to raised awareness, community buildup, and collaborations.

An example from recent times was seen during the COVID-19 pandemic. Global health, especially in terms of preparedness and response, has gathered tremendous attention and has become an important area of interest ever since. The pandemic reiterated the need for a cooperative, coordinated, global response to significant health challenges and the critical role that the field of global health plays in protecting and promoting the health of populations worldwide (2, 3). A multitude of this discourse was common on X during and after the pandemic by various stakeholders as well as the general public.

Despite recent ownership changes at X raising concerns within the scientific community about its potential impact on health research and communication (4, 5), its role in advocating for various health matters remains crucial. While many global health initiatives have made positive contributions to the healthcare domain, significant inequities persist in the real world, particularly in low-and middle-income countries (LMICs), which continue to face greater health challenges compared to high-income countries (HICs) (6). Addressing these disparities is essential to advancing global health and achieving equity for all populations. This study aims to understand the extent of such inequities on X in the discourse, dialog, and information sharing regarding #GlobalHealth.

The difference in public support for health systems in high-income countries (HICs) and low-and middle-income countries (LMICs) has previously been researched in studies that also highlight that the trajectory of health systems in LMICs will not meet the increasing demands for better health outcomes and greater social value (7). Therefore, substantial efforts are needed by LMICs to encourage more significant investments in health systems and initiatives that will provide their populations with the resources necessary to achieve better health outcomes. The concentration of global health organizations in the West, mainly Europe and North America, is primarily due to the historical development of the international health system and the concentration of wealth and resources in these regions. Due to this, these organizations have played a significant role in defining the global health agenda and determining the priorities and focus of global health initiatives (8). Consequently, solid actions need to be taken to boost the representation and involvement of LMICs in global health governance, discourse, and delivery.

Reflecting this in relation to the platform X, it is not known how wide the disparities in discourse are in the digital world. With this study, we aim to highlight the global distribution of posts, stakeholders, and influencers of #GlobalHealth. No previous studies have emphasized these digital discrepancies around #GlobalHealth on X.

## Materials and methods

The Symplur Signals research analytics tool was used to analyze #GlobalHealth posts for an extensive assessment of posts from 1st December 2019 to 1st November 2022. Symplur Signals is an analytical software that allows long-term tracking of posts containing specific hashtags pre-registered with the Symplur healthcare hashtag project (9). The analysis performed with Symplur Signals assessed the cumulative number of posts, impressions (i.e., views of posts), and user categorization into specific healthcare stakeholder groups. Other digital variable metrics included in the analysis were co-occurring hashtags, geolocation trends, and influencers. To understand the impact of influencers of the hashtag among the X community, the Symplur Rank system was used. For location data, the X profiles of each influencer were manually searched to evaluate and use information from the profile bios.

### Potential biases and limitations

Several potential biases and limitations are associated with using social media data for this analysis. One key limitation is the overrepresentation of certain demographic groups, as not all populations have equal access to social media platforms like X. This may lead to biased results that disproportionately reflect the views of more technologically connected groups. Secondly, only the posts in English language were analyzed. Additionally, using specific hashtags may not capture all relevant posts, as some users may discuss global health-related topics without specifically tagging the discussion with #GlobalHealth.

### Ethical considerations

While this study did not require ethical approval since it is based on pre-existing publicly available data, it is essential to address broader ethical considerations. Although the data set was anonymized and no personal information or identities were disclosed, there remain ethical implications when analyzing public social media content. Hence, careful consideration was given to ensure that no identifiable information was included and all data was handled in accordance with ethical standards for social media research.

## Results

The study has analyzed a large number of X posts (N = 843,762) posts identified using the #GlobalHealth hashtag and spanned between 1st December, 2019, and 1st November, 2022. These posts were shared by a massive 150,408 unique X users and generated 4,639,144,304 impressions (views), implying the extent of usage of the hashtag and its public popularity.

Among the content of the posts retrieved through Symplur Signals, some associating hashtags indicating the overlapping discourse and popular sub-topics with global health were also retrieved. The most common co-occurring hashtags with #globalhealth were #COVID19 (co-occurring in 42.8% of the posts), #Publichealth (32.6%), #analytics (30.2%), #COVID (29.6%), #datascience (29.1%), #data (28.7%), #datavisualisation (28.6%), #healthtech (19.5%), #health (13.5%) and #omicron (10.3%), listed in Table 1.

**Table 1:** Top co-occurring hashtags with #GlobalHealth (Popular associated topics with #GlobalHealth).

Co-occurring hashtags with #GlobalHealth		
	Hashtag	Percentage of co-occurrence (%)
1	#COVID19	42.8
2	#PublicHealth	32.6
3	#analytics	30.2
4	#COVID	29.6
5	#datascience	29.1
6	#data	28.7
7	#datavisualisation	28.6
8	#healthtech	19.5
9	#health	13.5
10	#omicron	10.3

**Figure 1:**
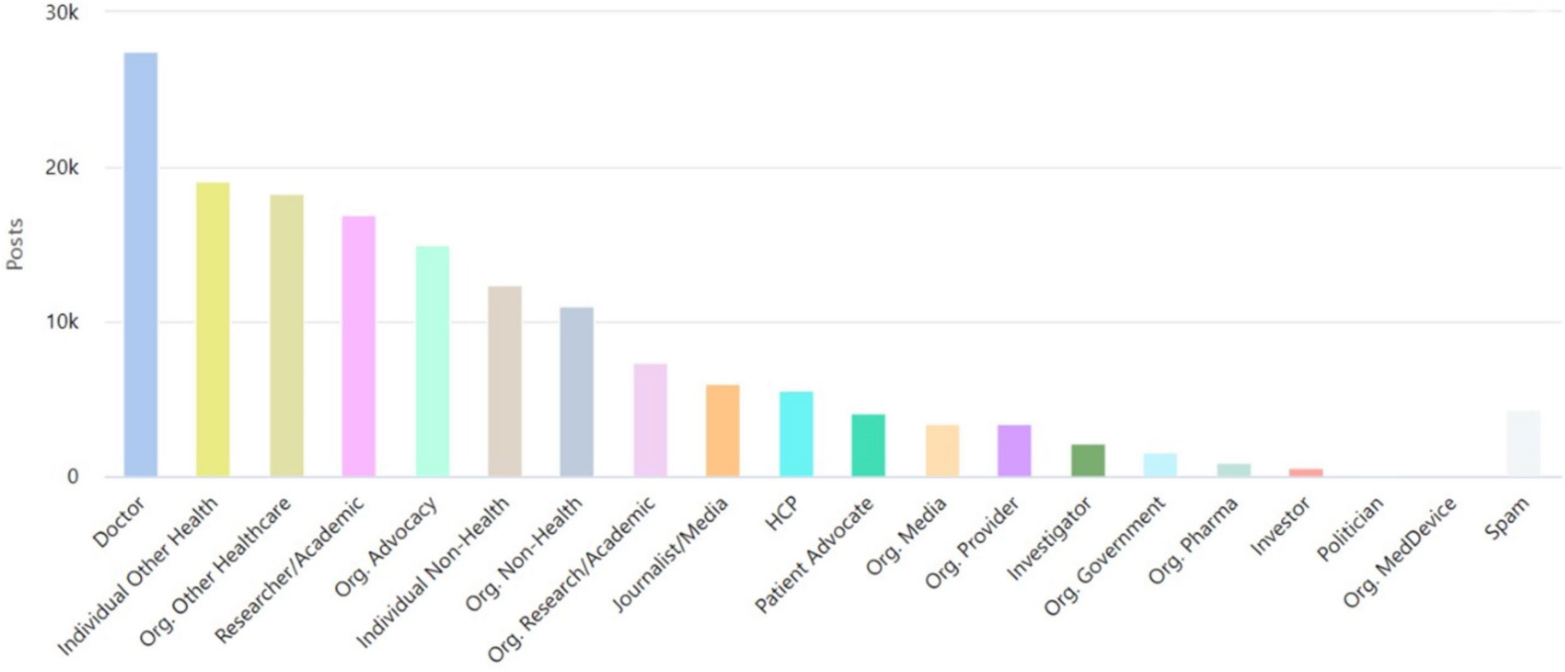
Top stakeholders using #GlobalHealth on X.

Analyzing the posts for stakeholder mapping (using Symplur Signals analysis of the available X bios of individual profiles), the five main stakeholders (Figure 1) were doctors (4.2%), followed by Individual other health workers (3.3%), Researchers/academicians (3.2%), Other healthcare workers (3.0%) and Org. Advocates (3.0%). It is essential to mention that most of the posts (73.8%) were made through accounts that could not be categorized in stakeholder categories and hence were flagged as “unknown.”

**Figure 2:**
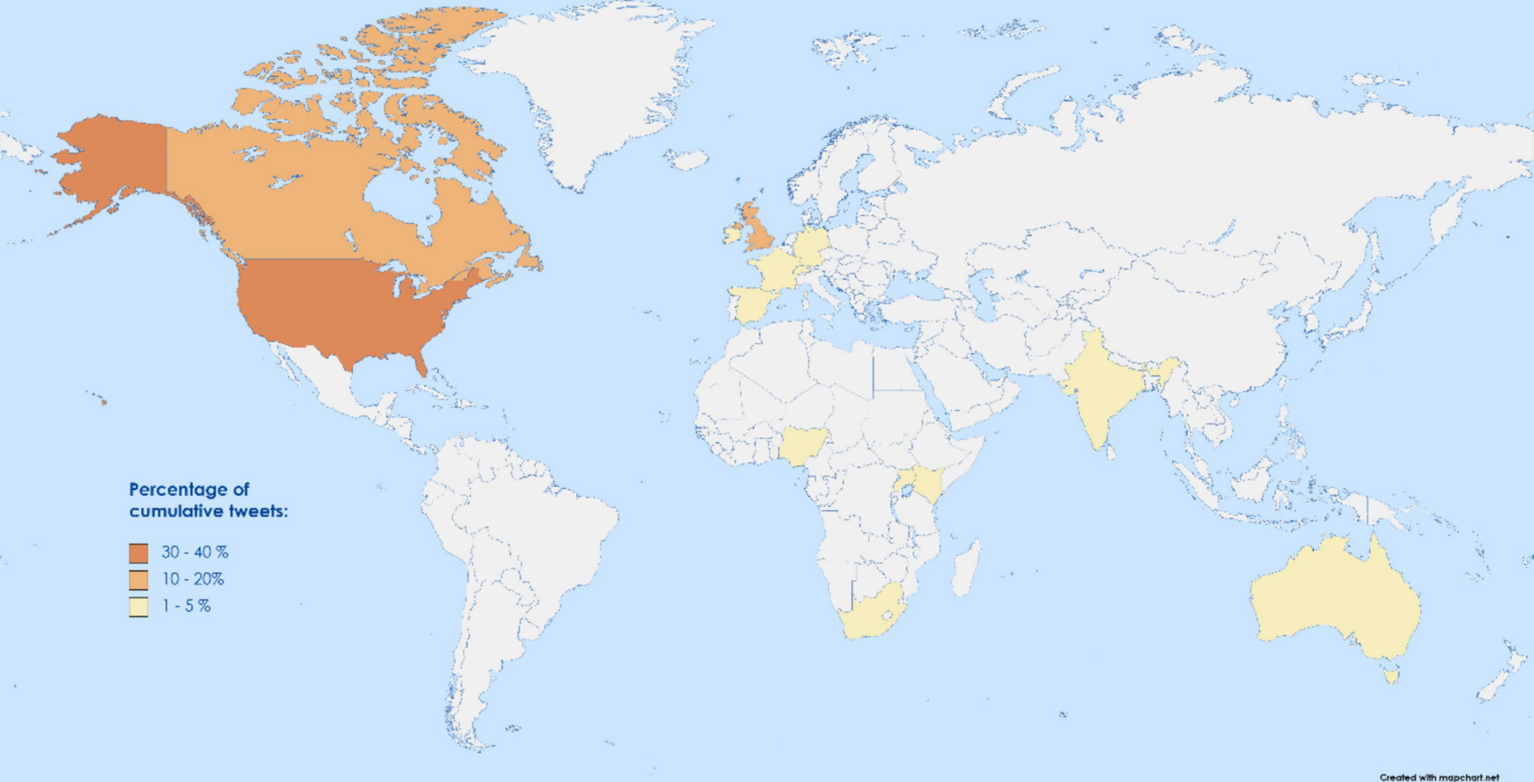
World map highlighting the global usage of #GlobalHealth on X in terms of frequency of posts.

Regarding the global distribution of #GlobalHealth, Figure 2 elaborates on the top 15 locations of the users according to the data provided in the users’ profiles. Accordingly, the most frequent users were from the United States (35.5%, *N* = 56.332) followed by the United Kingdom (15.9%, *N* = 25,265), Canada (12.2%, *N* = 19,328), India (4.0%, *N* = 6,365), Germany (2.8%, *N* = 4,461), Switzerland (2.8%, *N* = 4,415), Australia (2.5%, *N* = 4,041), Nigeria (1.9%, *N* = 3,119), Kenya (1.7%, *N* = 2,758), Spain (1.6%, *N* = 2,577), France (1.48%, *N* = 2,355), South Africa (1.30%, *N* = 2066), Netherlands (1.08%, *N* = 1,720), Uganda (0.98%, *N* = 1,563) and Ireland (0.79%, *N* = 1,256) in descending order suggesting an uneven global representation in #GlobalHealth discussions on X.

Further, with the data retrieved from Symplur Signals, some impactful influencers (personal accounts that specially had a higher engagement around their posts) of #GlobalHealth on X were ranked. The SymplurRank method was used to rank them, and their locations were traced back manually. Among the top 25 most influential handles, 9 were based in the United States, 6 in the United Kingdom, 3 in Canada, 3 in Switzerland, 1 in Australia, 1 in Germany, 1 in Dubai, and 1 in the Philippines. This further emphasized unequal global distribution. 10 profiles, each categorized as international organizations and individuals while 3 as journals, 1 as a medical school, and 1 as a media outlet.

## Discussion

This study about the modern applications of social media demonstrates a thorough analysis of the use of #GlobalHealth on the popular platform X in the target timeframe. The study extends over an approximately 3-year period, analyzing a very large number of posts, i.e., 843,762, using the #GlobalHealth hashtag by 150,408 unique X users. The data indicates the geolocations of the posts, the stakeholders engaging with #GlobalHealth, the popular influencers of the hashtag, and the engaging network mapping.

It is pertinent to note that although certain social media studies revolve around themes in the Global Health community of X (10–12), a hashtag analysis pinning to the location mapping of the posts with #GlobalHealth has not been put forward earlier.

In this study, the analysis of #GlobalHealth usage based on the location yielded significant results revealing geological discrepancies in the origin of the posts. Comprehending the current statistics on the general number of X users, the United States takes the lead among all countries, followed by Japan and India, in descending order (13). In our data, #GlobalHealth use comes the highest from the USA. In our data set, among the top 15 countries with the highest posts, 10 countries were high economic countries (HICs; Figure 2) that have, by default, infrastructurally better access to internet facilities, better digital literacy, a higher number of professionals and more advocates of global health itself (14–16). Even though, among the LMIC countries, India has the 4th highest frequency of posts, the difference in the percentage of posts (31%) is huge enough to emphasize the digital discrepancy of #GlobalHealth use. In the real world, in the last few years, researchers have explicitly been writing about the on-ground inequities in Global Health, not just regarding health delivery but also in terms of partnerships, funding, and capacity building (17, 18).

Likewise, the influencers of #globalhealth on X showed a similar pattern of geographical discrepancy. Among the top 25 most influential handles, all except 2 originated from High-income countries (HICs). 10 profiles, each categorized as international organizations with centers based in the global north, while 3 journal profiles also show their base from a HIC. This pattern mirrors previous literature, such as the work by Zenone et al., who discuss power imbalances in global health (19). Further studies have also expanded on the neo-colonialism that these patterns of discrepancy bring (20, 21), giving a global action call for decolonizing global health systems (22) decisions and governance (21, 23), ultimately aiding a more equitable and representative approach to global health governance and decision-making.

Further, although this study does not analyze gender discrepancies, gender-based disparities in global health representation are well-documented, echoing unequal representation in global health discussions, which is relevant to our findings on influencer dominance. Despite repeated commitments to gender equity, studies show that women remain underrepresented in high-level global health forums (24, 25). The dominance of HIC-based influencers in our analysis reflects the persistent structural inequities within global health leadership and digital influence.

Regarding stakeholder participation, ‘Doctors’ comprised the largest segment of classified users contributing to the #GlobalHealth discourse. Notably, ‘other health workers’ and ‘researchers/academics’, although outside the conventional healthcare system, were also significant contributors. However, a substantial portion of stakeholders remained unclassified. Similar stakeholder participation was identified in some previous studies, such as one from 2019 where clinicians and healthcare organizations made the top two #asthma tweeters over 4 years (26), and a 2022 study where analyses of #MedTwitterAI showed the highest number of posts from ‘healthcare workers’ followed by ‘doctors’ (27). Both studies encountered a large number of unknowns and non-classifiable users (48.56 percent) of the accounts labeled as “unknown” in the #MedTwitterAI study (27), which is similar to the present work. The lack of information on these users makes it difficult to categorize them. Furthermore, the previous studies noted that they could not manually verify every stakeholder classification, which could contribute to the uncertainty surrounding the unknown users. This highlights the importance of having clear and accurate information on social media profiles.

Among the most frequent co-occurring hashtags retrieved through the analysis, #COVID19 and #PublicHealth were the most frequent ones, which is self-explanatory due to the relevance of these terms during the COVID-19 Pandemic period. This also reflects how the globality of the pandemic brought forward various discourses related to COVID-19 and global health. Examples include the rise of COVID-19 vaccine opposition groups on X (28), bipolar sentiments across different regions about the disease (29), and the ‘infodemic’ phenomena (30) that were seen subsequently emphasizing the need for informed, cohesive responses to mitigate the challenges posed by the COVID-19 infodemic (30). The transdisciplinary impact of social media on health communication has become more pronounced post-pandemic, and further research could build upon the foundations established by this study to examine the evolving digital landscape and its implications for global health equity.

This study is the first to assess the distribution of #GlobalHealth users in terms of geographic representation, contributing novel insights into the digital inequities in global health discourse contributing to the emerging literature on the role of social media in global health discourse. The study uses large global data spread over an extended 3-year period, allowing credibility to reflect on the digital parameters discussed in the study. Further, the study focuses on geospatial analysis of user engagement, a dimension that had not been previously explored in relation to the #GlobalHealth hashtag. Despite its strengths, it is limited by its focus on X, and future studies should examine global health conversations across multiple social media platforms to gain a more comprehensive understanding of the dialog and themes of conversations. Furthermore, while our dataset captures a global audience, a significant portion of stakeholder contributors remained unclassified, limiting the study’s ability to categorize key influencers. Since this dataset analyzed only posts in English language, posts in other languages have not been represented in the analysis, in this respect future studies encompassing also non-English posts can be of value for gaining further insights. Sentiment analysis and a deeper dive into the content of individual posts were also beyond the scope of this study, leaving room for future research to explore these areas further.

Looking ahead to future research, this study paves paths for multiple interdisciplinary and mixed-method analysis to understand better the nature of global health conversations and discourse on social media. Deeper insight into sentiments and content of posts, along with differences in discourse on different social media platforms, could be explored to understand public sentiment informing future global health action and policy. At the same time, it is imperative for policy efforts to reduce digital disparities by promoting greater inclusion of LMIC voices in global health discourse, both online and offline. Future studies could also integrate broader media discussions on digital health communication and equity, particularly in the context of post-COVID-19 transformations in global health dialog and discussion.

## Conclusion

Our analysis of #GlobalHealth on X highlights significant digital discrepancies in geographical distribution, stakeholders, and influencers, mirroring real-world global health inequities. To bridge this gap, involving more diverse stakeholders, including patient advocates, policymakers, and the general public, in global health discussions is crucial. By promoting collaboration, information sharing, and wider access to digital tools, we can better represent global health concerns in the virtual space. Addressing these disparities will help reduce inequities in global health, allowing progress in research, practice, and global health delivery.

## Data Availability

The original contributions presented in the study are included in the article/supplementary material, further inquiries can be directed to the corresponding author.
