# Erratum: Is global health truly global? A hashtag analysis of #GlobalHealth disparities on X

**DOI:** 10.3389/fpubh.2025.1563573

**Published:** 2025-02-06

**Authors:** Frontiers Production Office

**Affiliations:** Frontiers Media SA, Lausanne, Switzerland

**Keywords:** global health, global health equity, global health discrepancy, hashtag, X, Twitter social media

Due to a production error, the correct funding statement was erroneously omitted. The funding statement should be:

“The author(s) declare financial support was received for the research, authorship, and/or publication of this article. The authors are grateful for the support of the Project POPUL/SP/0120/2023/01 directed by the Medical University of Warsaw.”

The publisher apologizes for this mistake. The original version of this article has been updated.

